# Supplementary material for: SelectMDx and Multiparametric Magnetic Resonance Imaging of the Prostate for Men Undergoing Primary Prostate Biopsy: A Prospective Assessment in a Multi-Institutional Study
Source: Cancers (Basel). 2021 Apr 23;13(9):2047. doi: 10.3390/cancers13092047 (PMC8122883; doi:10.3390/cancers13092047)
Supplement: Supplementary file 1 [file cancers-13-02047-s001.zip › cancers-1176547-supplementary.pdf]

cancers

MDPI

Supplementary

SelectMDx and Multiparametric Magnetic Resonance Imaging of the Prostate for Men Undergoing Primary Prostate Biopsy: A Prospective Assessment in a Multi-Institutional Study

Martina Maggi et. al.

Table S1. Patients characteristics (number, %, mean ± SD, median, range).

Parameter	Value
**Number of cases**, *n*	310
**Age** (years)	
mean± SD	64 ± 7.9
median	65
range	44–79
**Prostate volume** (mL)	
mean± SD	59.0 ± 23.9
median	56.0
range	21.0–131.0
**Total PSA** (*ng/mL*)	
mean± SD	7.6 ± 4.3
median,	6.6
range	1.0–19.9
**PSAD** (*ng/mL*)	
mean± SD	0.14 ± 0.09
median,	0.12
range	0.02–0.53
**DRE suspicious**, *n* (%)	
yes	76 (24.5)
no	234 (75.5)
**Family history**, *n* (%)	
yes	60 (19.3)
no	205 (80.7)
**SelectMDx score**, *n* (%)	
negative	166 (53.5)
positive	144 (46.5)
**mpMRI PI-RADS score**, *n* (%)	
PI-RADS 1–2	178 (57.4)
PI-RADS 3	54 (17.4)
PI-RADS 4–5	78 (25.2)
**SelectMDx score and mpMRI PI-RADS score**, *n (%)*	
SelectMDx positive, mpMRI positive	54 (17.5)
SelectMDx negative, mpMRI negative	142 (45.8)
SelectMDx positive, mpMRI negative	90 (29.0)
SelectMDx negative, mpMRI positive	24 (7.7)
**PCa at biopsy**, *n* (%)	104 (33.5)
**csPCa at biopsy**, *n* (%)	62 (20.0)

Abbreviations: *n* = number, SD = standard deviation, PSA = prostate-specific antigen, PSAD = PSA density, DRE = digital rectal examination, mpMRI = Multiparametric magnetic resonance imaging, PI-RADS = Prostate Imaging Reporting and Data System, PCa = prostate cancer, csPCa = clinically significant PCa.

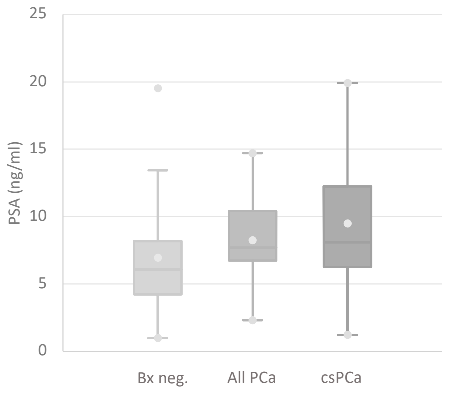

Figure S1a

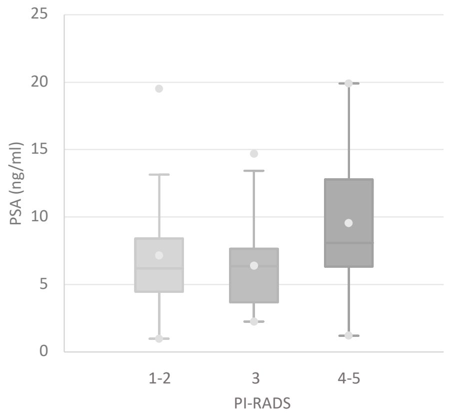

Figure S1b

Figure S1: (**a**) Total PSA in negative and positive PCa cases at biopsy; (**b**) Total PSA distribution according to PI-RADS score at mpMRI

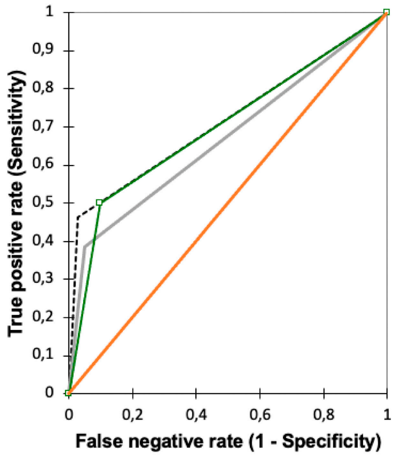

variable	*AUC*	*SE*	*95%CI*
**mpMRI + SelectMDx**	0.72	0.02	0.67-0.77
**mpMRI + PSA**	0.70	0.03	0.65-0.75
**mpMRI + PSAD**	0.67	0.02	0.62-0.71

Figure S2a

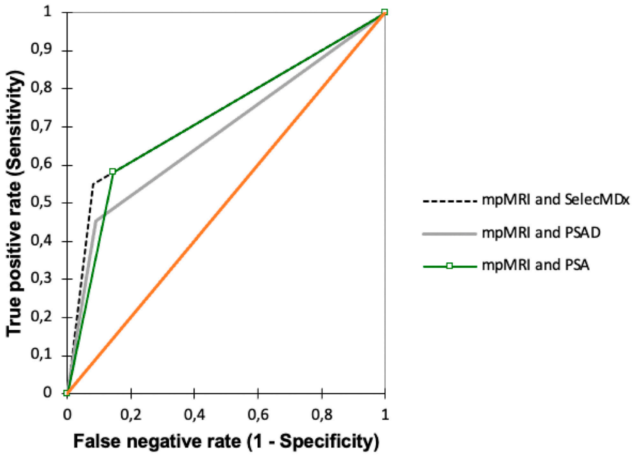

variable	*AUC*	*SE*	*95%CI*
**mpMRI + SelectMDx**	0.73	0.03	0.67-0.80
**mpMRI + PSA**	0.72	0.03	0.65-0.78
**mpMRI + PSAD**	0.68	0.03	0.62-0.75

Figure S2b

Figure S2: Performance of the association of mpMRI PI-RADS score with SelectMDx, PSA or PSAD evaluated as area under the curve (AUC) of the receiver operating characteristics (ROC) in predicting (**a**) PCa and (**b**) csPCa histological diagnosis at biopsy.

Table S2. Uni- and Multivariable Logistic Regression Model predicting variables.

(**a**) **Influencing Detection Rate of PCa**					
**Variables**		**Univariate analysis**		**Multivariate analysis***	
		HR (95%CI)	*p*-value	HR (95%CI)	*p*-value
Age, years	<65	Ref	--	--	--
	≥65	0.85 (0.49–1.49)	0.570	--	--
Age, years	Continuous	0.99 (0.95 – 1.02)	0.414	--	--
Prostate Volume, mL	<56	Ref	--	--	--
	≥56	0.77 (0.44–1.35)	0.364	--	--
Prostate Volume, mL	Continuous	1.00 (0.99–1.02)	0.476	--	--
PCa Familiarity	no	Ref	--	--	--
	yes	1.61 (0.84–3.11)	0.153	--	--
DRE	Negative	Ref	--	--	--
	Suspicious	1.09 (0.58–2.06)	0.792	--	--
Total PSA, ng/mL	Continuous	1.13 (1.06–1.20)	0.000	0.98 (0.90–1.08)	0.723
PSA density	<0.15	Ref	--	Ref	--
	≥0.15	3.26 (1.84–5.77)	< 0.0001	1.11 (0.45–2.71)	0.827
mpMRI PI-RADS	Negative (1–3)	Ref	--	Ref	--
	Positive (4–5)	8.23 (4.46–15.20)	< 0.0001	6.10 (2.94–12.64)	<0.0001
SelectMDx	Negative	Ref	--	Ref	--
	Positive	11.85 (5.40–26.01)	< 0.0001	9.49 (4.01–22.49)	<0.0001

(**b**) **Influencing Detection Rate of csPCa**					
**Variables**		**Univariate analysis**		**Multivariate analysis***	
		HR (95%CI)	*p*-value	HR (95%CI)	*p*-value
Age, years	<65	Ref	--	--	--
	≥65	1.01 (0.98–1.04)	0.617	--	--
Age, years	Continuous	1.14 (0.71–1.84)	0.576	--	--
Prostate Volume, mL	<56	Ref	--	--	--
	≥56	0.78 (0.48–1.25)	0.297	--	--
Prostate Volume, mL	Continuous	1.01 (0.97–1.05)	0.621	--	--
PCa Familiarity	no	Ref	--	Ref	--
	yes	2.38 (1.34–4.22)	0.003	4.49 (1.85–10.91)	0.001
DRE	Negative	Ref	--	Ref	--
	Suspicious	1.90 (1.11–3.23)	0.018	0.88 (0.42–1.84)	0.734
Total PSA, ng/mL	Continuous	1.12 (1.06–1.18)	0.001	0.97 (0.88–1.06)	0.466
PSA density	<0.15	Ref	--	Ref	--
	≥0.15	2.89 (1.77–4.73)	<0.0001	1.23 (0.51–2.97)	0.648
mpMRI PI-RADS	Negative (1–3)	Ref	--	Ref	--
	Positive (4–5)	8.19 (4.61–14.54)	<0.0001	7.14 (3.31–15.39)	<0.0001
SelectMDx	Negative	Ref	--	Ref	--
	Positive	18.10 (9.51–34.42)	<0.0001	25.57 (11.05–59.16)	<0.0001

Abbreviations: PCa = prostate cancer; csPCa = clinically significant PCa; HR = hazard ratio; DRE = digital rectal examination; PSA = prostate-specific antigen; mpMRI = multiparametric magnetic resonance imaging; PI-RADS = Prostate Imaging-Reporting and Data System.
